# Fear of progression in parents of childhood cancer survivors: prevalence and associated factors

**DOI:** 10.1007/s11764-021-01076-w

**Published:** 2021-07-23

**Authors:** Mona L. Peikert, Laura Inhestern, Konstantin A. Krauth, Gabriele Escherich, Stefan Rutkowski, Daniela Kandels, Louis J. Schiekiera, Corinna Bergelt

**Affiliations:** 1 Department of Medical Psychology, University Medical Center Hamburg-Eppendorf, Martinistraße 52, 20246 Hamburg, Germany; 2 Department of Pediatrics, Pediatric Hematology and Oncology, Klinik Bad Oexen, Oexen 27, 32549 Bad Oeynhausen, Germany; 3 Department of Pediatric Hematology and Oncology, University Medical Center Hamburg-Eppendorf, Martinistraße 52, 20246 Hamburg, Germany; 4 Swabian Children’s Cancer Center, Medical Faculty, University of Augsburg, Stenglinstraße 2, 86156 Augsburg, Germany

**Keywords:** Cancer, Fear, Parents, Pediatrics, Psycho-oncology, Survivors

## Abstract

**Purpose:**

Recent research demonstrated that fear of progression (FoP) is a major burden for adult cancer survivors. However, knowledge on FoP in parents of childhood cancer survivors is scarce. This study aimed to determine the proportion of parents who show dysfunctional levels of FoP, to investigate gender differences, and to examine factors associated with FoP in mothers and fathers.

**Methods:**

Five hundred sixteen parents of pediatric cancer survivors (aged 0–17 years at diagnosis of leukemia or central nervous system (CNS) tumor) were consecutively recruited after the end of intensive cancer treatment. We conducted hierarchical multiple regression analyses for mothers and fathers and integrated parent-, patient-, and family-related factors in the models.

**Results:**

Significantly more mothers (54%) than fathers (41%) suffered from dysfunctional levels of FoP. Maternal FoP was significantly associated with depression, a medical coping style, a child diagnosed with a CNS tumor in comparison to leukemia, and lower family functioning (adjusted *R*^*2*^ = .30, *p* < .001). Paternal FoP was significantly associated with a lower level of education, depression, a family coping style, a child diagnosed with a CNS tumor in comparison to leukemia, and fewer siblings (adjusted *R*^*2*^ = .48, *p* < .001).

**Conclusions:**

FoP represents a great burden for parents of pediatric cancer survivors. We identified associated factors of parental FoP. Some of these factors can be targeted by health care professionals within psychosocial interventions and others can provide an indication for an increased risk for higher levels of FoP.

**Implications for Cancer Survivors:**

Psychosocial support targeting FoP in parents of childhood cancer survivors is highly indicated.

## Introduction

More than 2000 children and adolescents in Germany under 18 years of age are diagnosed with cancer each year [1]. With a 15-year survival rate of approximately 80%, the population of childhood cancer survivors and their families is growing [1, 2]. Even though most parents adapt well after the end of their child’s cancer treatment [3, 4], some parents have to deal with long-term psychosocial burden (e.g., depression, anxiety, and posttraumatic stress) [3–6]. Whereas fear of progression (FoP), also referred to as fear of relapse or fear of cancer recurrence, in adult cancer patients has been intensively researched in the last decades [7, 8], only little is known about FoP in parents of childhood cancer survivors. FoP and fear of cancer recurrence or fear of relapse are distinct yet related constructs that share relevant aspects. FoP is defined as the “fear that the illness will progress with all its biopsychosocial consequences, or that it will recur” [9]. It describes a rationally explainable emotional response to a potentially life-threatening disease and is a generic concept of illness-related fears that is applicable on various chronic diseases with different courses (e.g., progression, recurrence) [9]. Therefore, we decided to use the term FoP in this study. FoP does not only concern the survivor, but also their caregivers [10, 11]. A study on FoP in adult cancer survivors and their caregivers found that caregivers reported even higher levels of FoP than survivors [11]. According to recent expert online survey in Germany, health care professionals frequently perceive parental FoP in their clinical practice [12]. Nevertheless, over a long period only qualitative studies described the phenomenon of parental FoP [4, 13–17]. Initial quantitative studies displayed that parental FoP seems to be associated with a low quality of life (QoL), depression, anxiety, and posttraumatic stress [18–20]. Previous studies delivered inconsistent results on associated factors of parental FoP. A study on parents of childhood cancer patients and long-term survivors found significantly negative correlations of FoP with time since diagnosis, parental age, current medical condition of the ill child, number of siblings, and parental anxiety coping skills [19]. This study did not reveal any significant gender differences in FoP levels of mothers and fathers and did not find a significant association between parental FoP and the child’s age at diagnosis [19]. The authors additionally calculated a multiple regression analysis and found significant associations of the child’s current medical condition and the parent’s anxiety coping skills with parental FoP [19]. Another study on parents of children with hematological cancer also did not find gender differences in FoP levels, but they also did not find an association with the time since diagnosis [21]. A recent study on fear of recurrence in adult couples with cancer displayed that male caregivers of women with cancer show higher levels of fear of cancer recurrence than female caregivers [22]. However, female cancer patients reported significantly higher levels of fear of cancer recurrence than male cancer patients.

Overall, little is known about FoP in parents of childhood cancer patients. Due to the small number of studies and the divergent results available, knowledge on factors associated with FoP in parents of childhood cancer survivors is scarce. The identification of associated factors could help health care professionals to identify parents at risk for suffering from dysfunctional levels of FoP. Furthermore, associated factors, for instance gender differences or differences between diagnosis groups with a different risk of progression [1], could provide approaches for targeted interventions and prevention strategies. Moreover, examining parental FoP is particularly relevant since it may also affect the child’s anxiety and distress [23]. This study aimed to determine the proportion of parents who show a dysfunctional level of FoP and to examine associated factors of FoP in mothers and fathers of childhood cancer survivors. Due to the limited data available, the choice of potentially associated factors of FoP follows a rather explorative approach and is based on the literature on FoP in adult patients and caregivers and our clinical experience [11, 22, 24, 25]. The research questions (RQ) are:

- RQ 1: What is the proportion of mothers and fathers of childhood cancer survivors who show a dysfunctional level of FoP?
- RQ 2: Are mothers or fathers prone to showing dysfunctional levels of FoP?
- RQ 3: Which parent-, patient-, and family-related factors are associated with FoP in mothers and fathers?

## Methods

### Design

This cross-sectional, questionnaire-based study is part of a prospective observational study with a longitudinal mixed-methods design. The overall study has been described in a study protocol [26].

### Participants and procedure

In this study, we focus on the most frequent pediatric cancers in Germany, leukemia and central nervous system (CNS) tumors [1]. We included biological parents and other caregivers of children under the age of 18 years at time of diagnosis. Parents were recruited after the end of intensive cancer treatment (e.g., radiation therapy, chemotherapy, surgery). At that point in time, patients in the German health care system switch into aftercare regardless of whether they receive maintenance treatment or not. Exclusion criteria were assessed by the health care providers in the clinics and included physical and/or mental burden (clinical decision by health care providers, applicable if the study participation would be overly burdensome), cognitive limitations, insufficient German language skills, and no interest. We consecutively recruited parents in Germany from July 2016 to March 2019 in two settings: (1) Study registries (International HIT-MED Registry, ClinicalTrials.gov Identifier: NCT02417324; COALL 08-09 study, ClinicalTrials.gov Identifier: NCT01228331; SIOP-LGG 2004 study, ClinicalTrials.gov Identifier: NCT00276640) informed the patients’ clinic after the end of intensive cancer treatment about the study. Health care providers in the clinics informed the parents about the study and provided a consent form to contact the family. If the family gave their consent to be contacted, the research institute sent the study material (written information, consent forms for study participation, and questionnaires) to the parents. (2) Our cooperating rehabilitation clinic informed the families at the beginning of an inpatient rehabilitation program about the study, obtained informed consent, and provided the study material to the families. Detailed information on the recruitment process is provided in the study protocol [26].

### Measures

#### Sociodemographic and medical data

Parents reported sociodemographic and medical information via questionnaire. Depending on the recruitment path, the diagnosis and time since diagnosis were extracted either from the parent’s report or the physician’s report in the rehabilitation clinic.

#### Fear of progression

FoP was measured with the 12-item Fear of Progression Questionnaire for the parental perspective (FoP-Q-SF/PR) [19]. The FoP-Q-SF/PR is an adaptation of the FoP-Q-SF for adult patients [27]. Parents report on a 5-point Likert scale from never (1) to very often (5) to which extent they feel burdened by aspects of FoP (e.g., I am concerned that my child will have to rely on outside help in everyday life). The FoP-Q-SF/PR allows the calculation of a sum score with higher scores indicating a higher level of FoP. Herschbach et al. [28] suggested a cut-off of ≥ 34 to differentiate functional from dysfunctional levels of FoP. The authors investigated a sample of adult cancer patients using the FoP-Q-SF and calculated the median score (*Md* = 34). In a second step, they stratified the sample according to their self-reported need for treatment and found a high degree of alignment [28]. This cut-off has also been used in an earlier study on FoP in parents of childhood cancer patients [21]. The FoP-Q-SF/PR has proved to be reliable and valid [18, 19]. Cronbach’s alpha was .86 in our sample.

#### Quality of life

Parental QoL was measured with The Ulm Quality of Life Inventory for Parents (ULQIE) [29]. The 29 items are rated on a 5-point Likert scale from never (0) to always (4). The ULQIE enables the calculation of both a total score and five subscale scores (functioning, satisfaction with family situation, emotional distress, self-development, and general well-being) with higher scores indicating a higher QoL. In this study, we used the total score. The ULQIE has adequate psychometric properties [29]. Cronbach’s alpha for the global scale was .93 in our sample.

Additionally, we used the 4-item physical well-being subscale of the KINDL-R [30, 31]. The KINDL-R was designed to assess children’s health-related QoL. Parents rate their child’s physical well-being of the past 7 days on a 5-point Likert scale from never (1) to all the time (5). The subscale can be transformed to a range of 0 to 100. Higher values indicate a higher physical well-being. The KINDL-R has proved to be reliable and valid [32, 33]. Cronbach’s alpha was .80 in our sample.

#### Depressive symptoms

Depressive symptoms in parents were measured with the 9-item depression module (PHQ-9) of the Patient Health Questionnaire (PHQ) [34]. The items have been constructed based on the diagnostic criteria for depression disorders of the Diagnostic and Statistical Manual of Mental Disorders (DSM-IV) [35]. The PHQ-9 uses a 4-point Likert scale ranging from not at all (0) to nearly every day (3). Higher sum scores indicate higher levels of depression. The PHQ-9 is a valid and reliable questionnaire [36, 37]. Cronbach’s alpha was .85 in our sample.

#### Coping

We measured parental coping with the Coping Health Inventory for Parents (CHIP) [38]. The 45 items cover three main coping patterns: family (CHIP_FAM: maintaining family integration, cooperation, optimistic definition of the situation), support (CHIP_SUP: maintaining social support, self-esteem, psychological stability), and medical (CHIP_MED: understanding the medical situation through communication with other parents and consultation with the medical staff) [39]. Answers are given on a 4-point Likert scale from not helpful (0) to extremely helpful (3). Additional options are “did not use” and “could not use.” Higher scores indicate helpful strategies. The CHIP has proved to be a valid and reliable instrument [39, 40]. In our sample, Cronbach’s alpha was .70 for the scale CHIP_FAM, .78 for the scale CHIP_SUP, and .70 for the scale CHIP_MED.

#### Family functioning

We measured family functioning by using the 12-item general functioning subscale of the McMaster Family Assessment Device (FAD-GF) [41]. Parents rate on a 4-point Likert scale from strongly agree (1) to strongly disagree (4) various aspects of family functioning (e.g., problem-solving behavior). A higher total score represents lower family functioning [41]. The FAD-GF is a reliable and valid instrument [42, 43]. Cronbach’s alpha was .86 in our sample.

### Statistical analyses

To determine the proportion of mothers and fathers who show a dysfunctional level of FoP, we utilized the cut-off of ≥ 34 suggested by Herschbach et al. [28]. We used Chi^2^ tests to investigate differences between mothers and fathers. Lastly, we conducted a hierarchical multiple regression analysis for mothers and fathers separately to identify factors associated with FoP. We decided for separate models to avoid a violation of the independence assumption in significance testing and to identify specific associated factors of FoP in mothers and fathers. Firstly, we included only parent-related factors (age, relationship status, education, employment status, quality of life, depression, coping pattern). Secondly, patient-related factors were added (age, gender, diagnosis, time since diagnosis, other chronic diseases or impairments, physical well-being). Lastly, we included family-related factors (family functioning, number of siblings). Dummy-coded variables were utilized when necessary. We tested the Gauss-Markov assumptions and modified the models accordingly. Additionally, we used unpaired t-tests to calculate gender differences in various psychosocial outcomes. The analyses were performed using the software *IBM SPSS Statistics 27*. Alpha was set at .05 for all analyses. Missing values in validated measures were imputed with the individual mean with a maximum of 30% missing data within the scale.

## Results

### Sample characteristics

Eight hundred ninety-nine families were potentially eligible for participation in the study. In total, 312 families participated in the survey (initial participation rate: 35%). Sixty families that were recruited via the rehabilitation clinic did not participate for the following reasons: No interest (*n* = 21), insufficient German language skills (*n* = 14), physical and/or mental burden (*n* = 12), cognitive limitations (*n* = 3), not specified (*n* = 10). The remaining 527 families that did not participate were recruited via the study registries. They either did not participate because they fulfilled the exclusion criteria or the health care providers in the clinics were not able to inform them about the study. From the 312 families that participated, five families were excluded from the analyses subsequently due to missing consent forms for participation (*n* = 2), a wrong diagnosis (*n* = 2), or incorrectly answered questionnaires because of limited German language skills (*n* = 1). In two families, only the children answered questionnaires. Hence, in this study, we analyzed the data of 516 parents of 305 families (Table 1). In 211 families, both parents participated. One hundred thirty-one of the families were recruited via the study registries and 174 in the rehabilitation clinic. There were no significant differences in FoP levels between parents in the two recruitment paths (*t*(511) = 0.289, *p* = .773).

**Table 1:** Sociodemographic and medical data of 516 parents and 305 pediatric cancer patients

Sociodemographic data						
**Parents**	**Total (*n*** **= 516)**		**Fathers (*n*** **= 217)**		**Mothers (*n*** **= 299)**	
	***M***	***SD*/range**	***M***	***SD*/range**	***M***	***SD*/range**
Age in years ^a^	39.4	7.3/20-70	41.1	7.6/23-70	38.2	6.9/20-64
	**n**	**%**	**n**	**%**	**n**	**%**
Permanent relationship	472	91.5	209	96.3	263	88.0
Education ^b^						
> 10 years	248	50.4	106	52.0	142	49.3
≤ 10 years	244	49.6	98	48.0	146	50.7
Employment status ^c^						
Gainfully employed	365	72.1	197	92.5	168	57.3
Full-time	208	57.0	177	89.8	31	18.5
Part-time	157	43.0	20	10.2	137	81.5
Not gainfully employed	104	20.6	11	5.2	93	31.7
Homemakers	65	62.5	1	9.1	64	68.8
(Re)Training	4	3.8	0	0	4	4.3
Seeking employment	30	28.8	7	63.6	23	24.7
Retired	5	4.8	3	27.3	2	2.2
Other, e.g., parental leave	37	7.3	5	2.3	32	10.9
**Patients**	**Total (*n*** **= 305)**		**Boys (*n*** **= 170)**		**Girls (*n*** **= 135)**	
	***M***	***SD*/range**	***M***	***SD*/range**	***M***	***SD*/range**
Age in years	7.3	4.3/1-18	7.9	4.5/1-18	6.6	4.0/1-17
Time since diagnosis in months	22.1	21.8/5-178	22.1	21.3/5-152	22.2	22.5/5-178
	**n**	**%**	**n**	**%**	**n**	**%**
Number of siblings						
0	59	19.3	29	17.1	30	22.2
1–2	222	72.8	124	72.9	98	72.6
> 2	24	7.9	17	10.0	7	5.2
Cancer diagnosis						
CNS tumor	157	51.5	89	52.4	68	50.4
Leukemia	148	48.5	81	47.6	67	49.6
Other chronic diseases or impairments, e.g., epilepsy, hemiparesis ^d^	82	27.0	46	27.2	36	26.7

^a^2 missings, ^b^24 missings, ^c^10 missings, ^d^1 missing

### Descriptive findings and gender differences

Fifty-four percent of the mothers (*n* = 161) and 41% of the fathers (*n* = 87) showed dysfunctional levels of FoP (Table 2). Significantly more mothers than fathers suffered from dysfunctional FoP levels (*Chi*^*2*^ = 8.692, *p* = .003). In the overall sample, the mean FoP sum score was *M* = 33.8 (*SD* = 9.4). Mothers reported significantly higher FoP and depression levels and a significantly lower QoL than fathers. We also found significant differences between mothers and fathers in coping patterns. The rating of the family functioning did not differ significantly between mothers and fathers. Looking at the parents’ rating of the patients’ physical well-being, there is no striking difference between mothers and fathers. We did not calculate gender differences for this variable to avoid a violation of the independence assumption since some parents rated the physical well-being of the same child.

**Table 2:** Descriptive data and gender differences

	Total (*n* = 516)		Fathers (*n* = 217)		Mothers (*n* = 299)			
**Parents** ^a^	***M***	***SD*/range**	***M***	***SD*/range**	***M***	***SD*/range**	***t***	***p***
Fear of progression (FoP-Q-SF/PR)	33.8	9.4/13–60	32.3	9.7/14–58	34.9	9.0/13–60	− 3.103	**.002**
Quality of life (ULQIE)	66.6	17.4/26–109	69.6	17.4/27–109	64.4	17.2/26–107	3.336	**.001**
Depression (PHQ-9)	7.4	5.2/0–25	6.2	4.9/0–23	8.2	5.2/0–25	− 4.418	**<.001**
Coping (CHIP)								
Family coping (CHIP_FAM)	44.0	6.2/10–57	43.7	6.8/10–57	44.1	5.7/23–57	− 0.693	.489
Support coping (CHIP_SUP)	29.1	8.0/3–51	27.9	8.0/3–51	30.0	7.9/9–50	− 2.521	**.012**
Medical coping (CHIP-MED)	15.8	4.2/1–24	15.0	4.3/1–24	16.4	4.0/4–24	− 3.853	**<.001**
Family dysfunction (FAD-GF)	1.8	0.6/1–4	1.8	0.6/1–4	1.8	0.6/1–4	− 0.912	.362
Fear of progression cut-off ^b^	***n***	**%**	***n***	**%**	***n***	**%**	**χ**^**2**^	***p***
Dysfunctional	248	48.3	87	40.7	161	53.8	8.692	**.003**
**Patients** ^a^	***M***	***SD*/range**	***M***	***SD*/range**	***M***	***SD*/range**		
Physical well-being (KINDL-R subscale)	67.3	21.4/0–100	67.4	22.1/13–100	67.2	21.0/0–100		

^a^FoP-Q-SF/PR 3 missings, ULQIE 5 missings, PHQ-9 4 missings, CHIP_FAM 35 missings, CHIP_SUP 141 missings, CHIP_MED 35 missings (additional options “did not use” and “could not use” were treated as missings), FAD_GF 9 missings, KINDL-R physical well-being subscale 15 missings

^b^FoP-Q-SF/PR sum scores ≥34 were considered dysfunctional levels of FoP

### Associated factors of parental FoP

The hierarchical multiple regression analyses were conducted using data of 295 mothers and 217 fathers of 305 families. Four mothers were excluded from the analyses since in four families two female caregivers participated. After a listwise deletion of missing data, the data of 213 mothers and 171 fathers was analyzed in the regression models. There were no significant differences in the FoP levels of mothers and fathers who were included in the regression analysis and those who were excluded due to missing values (mothers: *t*(293) = − 0.493, *p* = .622; fathers: *t*(212) = 0.559, *p* = .577). The variable relationship status was excluded for both mothers and fathers because of its low variance in our sample (96% of the fathers and 88% of the mothers in permanent relationship). The variable employment status was only excluded for fathers (93% gainfully employed). We also excluded the variable support coping (CHIP_SUP) due to a high number of missing values (Table 2). Lastly, we removed variables with an intercorrelation of *r* > .600 to avoid multicollinearity. Thus, QoL was excluded due to its high correlation with depression (mothers: *r* = − .731, *p* < .001; fathers: *r* = − .763, *p* < .001).

In the subsample of mothers, the parent-related factors in model 1 explained 21% of the variance in FoP (Table 3). Model 2 included parent- and patient-related factors and explained 26% of the variance in FoP. Model 3 incorporated parent-, patient-, and family-related factors and accounted for 30% of the variance in FoP. Integrating parent-related, patient-related, and family-related factors thus led to the highest predictive power. In model 3, depression, a medical coping style, and family dysfunction were associated with higher levels of FoP. Furthermore, a leukemia diagnosis of the child in comparison to a CNS tumor diagnosis was associated with lower levels of maternal FoP. In the subsample of fathers, model 1 explained 42% of the variance in FoP, model 2 explained 46% of the variance, and model 3 accounted for 48% of the variance in FoP (Table 4). In model 3, five factors were significantly associated with paternal FoP. Depression and a family coping style were associated with higher levels of paternal FoP. Additionally, a leukemia diagnosis of the child in comparison to a CNS tumor diagnosis, a higher level of education, and a higher number of siblings of the survivor were associated with lower levels of FoP in fathers.

**Table 3:** Hierarchical multiple regression of fear of progression with 213 mothers of childhood cancer survivors

	Model 1					Model 2					Model 3				
	*B*	*SE*	Beta	*T*	*p*	*B*	*SE*	Beta	*T*	*p*	*B*	*SE*	Beta	*T*	*p*
Parent-related factors															
Age	− 0.078	0.082	− .059	− 0.944	.346	− 0.159	0.095	− .121	− 1.680	.094	− 0.155	0.096	− .118	− 1.620	.107
Education ^a^	− 2.304	1.144	− .127	− 2.014	.045*	− 1.988	1.145	− .110	− 1.736	.084	− 2.172	1.120	− .120	− 1.939	.054
Employment status ^b^	0.995	1.233	.052	0.807	.421	1.004	1.206	.053	0.832	.406	0.506	1.198	.026	0.422	.673
Depression	0.721	0.110	.411	6.561	<.001*	0.697	0.113	.397	6.156	<.001*	0.562	0.118	.320	4.775	<.001*
Family coping	0.032	0.104	.020	0.303	.762	0.027	0.103	.017	0.264	.792	0.098	0.103	.062	0.951	.343
Medical coping	0.418	0.151	.184	2.774	.006*	0.421	0.147	.185	2.862	.005*	0.395	0.144	.174	2.748	.007
Patient-related factors															
Age						0.083	0.159	.041	0.523	.602	0.079	0.156	.039	0.508	.612
Gender ^c^						0.311	1.132	.017	0.275	.784	0.024	1.112	.001	0.022	.983
Diagnosis ^d^						− 2.918	1.180	− .161	− 2.473	.014*	− 3.723	1.180	− .205	− 3.156	.002*
Time since diagnosis						0.042	0.024	.110	1.710	.089	0.040	0.024	.105	1.661	.098
Other chronic diseases or impairments ^e^						0.716	1.344	.036	0.533	.595	0.935	1.315	.047	0.711	.478
Physical well-being						− 0.037	0.028	− .086	− 1.312	.191	− 0.025	0.028	− .059	− 0.912	.363
Family-related factors															
Family dysfunction											3.515	1.196	.194	2.938	.004*
Number of siblings											− 1.064	0.593	− .113	− 1.795	.074
*R*^*2*^ (adjusted *R*^*2*^)					.235 (.213)					.305 (.263)					.343 (.297)
*F*					10.559					7.305					7.398
*p*					<.001*					<.001*					<.001*
*R*^*2*^ change					.235					.070					.039
*F* change					10.559					3.333					5.840
*p* of *F* change					<.001*					.004*					.003*

^a^Reference category: >10 years, ^b^reference category: gainfully employed, ^c^reference category: female, ^d^reference category: leukemia, ^e^reference category: yes. *p < .05

**Table 4:** Hierarchical multiple regression of fear of progression with 171 fathers of childhood cancer survivors

	Model 1					Model 2					Model 3				
	*B*	*SE*	Beta	*T*	*p*	*B*	*SE*	Beta	*T*	*p*	*B*	*SE*	Beta	*T*	*p*
Parent-related factors															
Age	0.061	0.078	.047	0.786	.433	− 0.045	0.091	− .035	− 0.498	.619	− 0.005	0.091	− .004	− 0.059	.953
Education ^a^	− 3.918	1.185	− .202	− 3.306	.001*	− 4.204	1.181	− .217	− 3.558	<.001*	− 4.111	1.159	− .212	− 3.546	.001*
Depression	1.215	0.121	.590	10.039	<.001*	1.129	0.123	.549	9.173	<.001*	1.059	0.129	.515	8.232	<.001*
Family coping	0.186	0.102	.128	1.818	.071	0.202	0.100	.139	2.014	.046*	0.238	0.103	.164	2.309	.022*
Medical coping	0.104	0.161	.045	0.646	.519	0.072	0.159	.031	0.449	.654	0.106	0.157	.046	0.678	.499
Patient-related factors															
Age						0.133	0.160	.059	0.834	.406	0.171	0.160	.076	1.071	.286
Gender ^b^						− 0.822	1.165	− .042	− 0.705	.482	− 1.005	1.147	− .051	− 0.876	.382
Diagnosis ^c^						− 2.940	1.203	− .152	− 2.444	.016*	− 3.548	1.200	− .183	− 2.957	.004*
Time since diagnosis						0.054	0.032	.104	1.687	.093	0.044	0.032	.084	1.382	.169
Other chronic diseases or impairments ^d^						− 1.345	1.368	− .060	− 0.983	.327	− 1.614	1.345	− .072	− 1.200	.232
Physical well-being						− 0.032	0.027	− .072	− 1.170	.244	− 0.030	0.027	− .068	− 1.135	.258
Family-related factors															
Family dysfunction											1.986	1.275	.101	1.557	.122
Number of siblings											− 1.566	0.659	− .142	− 2.378	.019*
*R*^*2*^ (adjusted *R*^*2*^)					.436 (.419)					.492 (.456)					.517 (.477)
*F*					25.496					13.972					12.916
*p*					<.001*					<.001*					<.001*
*R*^*2*^ change					.436					.056					.025
*F* change					25.496					2.900					4.107
*p* of *F* change					<.001*					.010*					.018*

^a^Reference category: >10 years, ^b^reference category: female, ^c^reference category: leukemia, ^d^reference category: yes. *p < .05

## Discussion

In this study, we determined the proportion of parents of childhood cancer survivors who show dysfunctional levels of parental FoP, analyzed gender differences, and examined associated factors of FoP in mothers and fathers.

In the overall sample, 48% of the parents reported dysfunctional levels of FoP approximately 22 months after their child’s diagnosis. Significantly more mothers than fathers suffered from dysfunctional FoP. Even though earlier studies on parental FoP that used the FoP-Q-SF/PR did not find any gender differences [19, 21], mothers reporting higher levels of psychosocial burden than fathers is a common finding in psycho-oncological research [44, 45]. Some studies on adult cancer survivors also suggest that women experience higher levels of FoP [7]. The mean FoP score of the participating parents is comparable to or lower than FoP scores in earlier studies on parents of childhood cancer patients and survivors using the same instrument [18, 19, 21]. The sample sizes in these studies were considerably smaller than in the present study. The parents in our study reported comparable or significantly higher levels of FoP than adult cancer survivors in earlier studies [24, 46, 47] and similar FoP scores to those obtained in partners of adult cancer patients [48]. Notably, the comparability of our results with earlier studies is limited due to different measurement time points.

Regarding associated factors of FoP, our results indicate that the highest predictive power was achieved by integrating parent-, patient-, and family-related factors in the regression models. Thereby, the predictive power of the model was considerably higher in fathers than in mothers. Other factors than the ones that were assessed in this study (e.g., posttraumatic stress, general anxiety) might be associated especially with maternal FoP and should be investigated in future studies. Maternal FoP was significantly associated with a higher level of depression, greater benefit from a medical coping style (e.g., reading more about the medical problem which concerns me), a child diagnosed with a CNS tumor in comparison to leukemia, and lower family functioning. Paternal FoP was significantly associated with a lower level of education, a higher level of depression, greater benefit from a family-oriented coping style (e.g., investing myself in my child(ren), trying to maintain family stability), a CNS tumor diagnosis in comparison to a leukemia diagnosis, and the child having fewer siblings. Overall, FoP was more strongly associated with psychosocial variables than with sociodemographic variables. However, previous studies on adult cancer patients also found a significant association between lower levels of education and higher levels of FoP [8]. The association between depression and FoP was also found in earlier studies on adult cancer patients [24, 25, 46]. This association might occur because of a strong tendency of people with symptoms of depression to ruminate and worry. The association between coping and FoP has been investigated with a dyadic data analysis approach in a study with 44 parental couples, which found a significantly negative association between family and support coping patterns and FoP for mothers but not for fathers [21]. In contrast, in our study the family coping pattern was associated with higher FoP levels in fathers. Further research is necessary for a better comprehension of the association between coping and FoP. A CNS tumor diagnosis in a child seems to be a risk factor for higher FoP levels in comparison to a leukemia diagnosis. This result might be related to the higher risk of progression in CNS tumor survivors [1]. Significant family-related protective factors of parental FoP are a higher family functioning for mothers and a higher number of siblings for fathers. This result is in accordance with the findings of a recent interview study with parents of childhood cancer survivors [6]. Parents reported that the family and especially the siblings are an important resource when it comes to reintegration into family life after the end of intensive cancer treatment because they claim for normality [6].

### Clinical implications

Approximately one-half of the surveyed parents experienced dysfunctional levels of FoP after the end of their child’s intensive treatment. Parental FoP levels seem to be comparable to or even higher than FoP levels of adult cancer patients. Thus, psychosocial support programs targeting parental FoP are highly indicated. It should be noted that the cut-off used in this study is primarily based on statistical considerations in a sample of adult cancer patients. A recent study suggested characteristics of clinical levels of FoP [49]. A cut-off score based on clinical considerations could help health care professionals to identify parents that are in need of professional support. Initial studies on psychotherapeutic treatment approaches for FoP already exist for adult cancer patients but not for parents of childhood cancer survivors [9, 50]. In the present study, we identified associated factors of FoP in mothers and fathers. Whereas some of these factors could be targeted by health care professionals within psychosocial interventions (depression, coping, family functioning), others can provide an indication for an increased risk for higher levels of parental FoP (low level of education, CNS tumor diagnosis, fewer siblings).

Furthermore, the examination of the relationship between FoP in parents and survivors could provide further insights into FoP in families affected by childhood cancer.

### Study limitations

We recruited parents via study registries and a rehabilitation clinic, because a personal contacting of the parents was not possible due to reasons of data protection regulations. A systematic non-responder analysis could not be conducted within this study design and thus, the generalizability of the results might be limited. Furthermore, an underreporting of FoP is possible since we excluded parents with particularly high levels of mental burden for ethical reasons. Additionally, it is likely that some survivors still received maintenance treatment at the time of the survey to ensure the success of the initial cancer treatment which may have affected our results. In our clinical experience, the maintenance treatment gives parents a subjective feeling of security which could lead to a lower level of FoP. Still, an opposite effect is conceivable. Lastly, due to the limited data available on FoP in parents of childhood cancer survivors, the choice of potentially associated factors of FoP was explorative and relevant factors (e.g., posttraumatic stress) might have been missed. Race and ethnicity were not measured in this study and also might constitute important risk factors of FoP. However, this study has also several strengths. We have surveyed a large nationwide sample, including both mothers and fathers, with validated questionnaires and have delivered results in a research field that is still largely unexplored. Furthermore, the STROBE Statement [51] was used for the dissemination of the results.

### Conclusions

Our findings showed that a substantial proportion of parents of childhood cancer survivors report dysfunctional levels of FoP after the end of their child’s intensive treatment. Especially mothers are prone to suffer from dysfunctional FoP. As we identified depression, coping patterns, and family functioning as factors associated with FoP in mothers and fathers, these factors could be targeted in psychosocial interventions or prevention strategies. Additionally, health care professionals should pay attention to parents that may have a higher risk of suffering from FoP (low level of education, CNS tumor diagnosis of the child, survivors having fewer siblings).
